# Effort and Substance Use: Differentiating Tobacco Use Through Reinforcement Learning of Effort Based Decision Making

**DOI:** 10.21203/rs.3.rs-8928184/v1

**Published:** 2026-03-17

**Authors:** Kasey P. Spry, Jazmyne James, Alison H. Oliveto, Michael Mancino, Kenneth T. Kishida, Merideth A. Addicott

**Affiliations:** 1 Department of Translational Neuroscience, Wake Forest University School of Medicine, Winston-Salem, NC, USA; 2 Neuroscience Graduate Program, Wake Forest University Graduate School of Arts and Sciences, Winston-Salem, NC, USA; 3 Department of Psychiatry , University of Arkansas for Medical Sciences, Little Rock, AR, USA; 4 Department of Biology, Wake Forest University, Winston-Salem, NC, USA; 5 Department of Neurosurgery, Wake Forest University School of Medicine, Winston-Salem, NC, USA

**Keywords:** Effort, Tobacco, Opioids, Reinforcement learning, smoking, decision making

## Abstract

**BACKGROUND:**

Effort-based decision making evaluates rewards relative to the effort required to obtain it, an important process of healthy goal-directed motivation and behavior. Computational models provide mechanistic insights underlying choice behavior and potential alterations in neuropsychiatric disorders, including substance use disorders. We applied computational models to effort-based choice behavior to characterize underlying decision processes and if these mechanisms differ by substance use status.

**METHODS:**

Participants completed the Effort Expenditure for Rewards Task, choosing between low- and high-effort options for monetary rewards varying in magnitude and probability. Participants met criteria for no tobacco use (n = 23), current tobacco use disorder (n = 26), former tobacco use disorder (n = 22), and tobacco and opioid use disorder (n = 29). Computational models from two families, Subjective Value and Reinforcement Learning, were fit and compared. Parameters from the best-fitting model underwent principal components analysis and linear discriminant analysis.

**RESULTS:**

Temporal difference reinforcement learning model demonstrated greater model evidence and predictive accuracy, indicating better fit to effort-based choice behavior. Principal components analysis revealed meaningful multivariate distinctions: PC1 differentiated all groups except individuals without tobacco use versus individuals with tobacco use disorder; PC3 distinguished tobacco and opioid use disorder from all other groups. Linear discriminant analysis demonstrated group separation with 76% classification accuracy.

**CONCLUSIONS:**

A reinforcement learning framework better explained participants’ effort-based choice behavior. Substance use status relates to dynamic behavioral changes (i.e. learning) as measured by the multivariate combination of learning rate, future discounting, and choice temperature.

## INTRODUCTION

An important feature of healthy decision-making is the willingness to exert effort, either physical or cognitive effort, to obtain rewards. Effort-based decision making (EBDM) evaluates the value (i.e. magnitude and probability) of a reward in relation to the effort required to obtain it (Salamone et al., 2016). If effort expenditure is perceived as increasingly costly, the reward may become devalued (Inzlicht et al., 2018; Klein-Flügge et al., 2015). Cost-benefit analyses are an important process of goal-directed motivation and behavior, which allows individuals to obtain goals despite obstacles in the environment (Bailey et al., 2016).

Traditionally goal-directed behaviors are analyzed with generalized estimating equations or other repeated measures analyses to understand *what* choices are made (e.g. proportion of high/low effort option selections) and how this differs by diagnosis or group classification. There has been an increased focus on developing mechanistic accounts of the computational processes underlying goal-directed motivation and behavior, specifically EBDM (Huys et al., 2016). For example, computational models have been applied to EBDM tasks to investigate *how* choices are made (e.g. with or without systematic utilization of given information, learning from previous outcomes, etc.) (Cooper et al., 2019; Jarvis et al., 2022; Kuhn et al., 2025; Whitton et al., 2024). Cooper et. al (2019) used a computational approach on an EBDM task, the Effort Expenditure for Rewards Task (EEfRT), using models that vary in terms of their utilization of trial-wise information (reward magnitude, reward probability, and effort allocation) to guide choice behavior. While these types of models offer insight into how individuals use reward and effort information, they might not capture key details such as the influence of previous choices or outcomes (i.e., learning). This is an important limitation of these models because previous choices and outcomes influence succeeding choice behavior via reinforcement learning mechanisms (O’Doherty et al., 2003; Schultz et al., 1997; Sutton & Barto, 1998; Watkins & Dayan, 1992).

Reinforcement learning (RL) models are defined by the ability to learn from interactions with an environment through trial and error to maximize cumulative reward outcomes. One class of RL models, Rescorla-Wagner reinforcement learning models (defined by their ability to model learning as changes in associative strength between cues and outcomes), have previously applied to an EBDM paradigm, which combined a probabilistic reward task with physical effort. This model showed that effort results in more efficient learning from positive outcomes and less efficient learning from negative outcomes. This effect varies across individuals and is more pronounced in those who are more averse to effort at baseline (Jarvis et al., 2022). Another class of RL models are temporal difference reinforcement learning (TDRL) models, defined by their ability to learn predictions of future rewards over time by updating value estimates based on differences between successive predictions. To our knowledge, TDRL models have not been applied to EBDM paradigms. However, there is clear evidence that TDRL models track decision-making behavior and hypothesized underlying dopaminergic mechanisms. Specifically, TDRL tracks activity of dopamine (DA) neurons in the midbrain where TD errors are encoded by DA neurons (Hart et al., 2014; Liebenow et al., 2022; Montague et al., 1996; Sands, Jiang, Jones, et al., 2023; Sands, Jiang, Liebenow, et al., 2023; Schultz et al., 1997; Sutton & Barto, 1998). Thus, TDRL models may help clarify processes underlying decision-making behavior modulated by changes in DA signals, including EBDM. To this end, we investigated effort-based choice behavior with subjective value and reinforcement learning models to better understand *how* individuals are making effort-based choices and if these mechanisms differ by group classification, here substance use status.

In this report, we are using data published previously comparing EEfRT performance among participants with current, former, and never tobacco use (Addicott et al., 2020). These data were previously analyzed using generalized estimating equations. The analysis of computationally modeled EEfRT choice behavior in these groups is not simply a re-analyzation of existing work (i.e. sensitivity of groups to given information) but instead offers insights into group behavior in the use of given information to drive choice behavior between the two options. In essence, our focus here is to determine *how* individuals made EBDM choices as opposed to *what* EBDM choices were made. We also expanded this dataset to include individuals with both OUD and TUD who completed the EEfRT as part of a different study (R01 DA039088 MPI: Oliveto & Mancino). Over 80% of individuals with opioid use disorder (iOUD) use tobacco; however, dual use is rarely studied (John et al., 2019) and the specific effects of tobacco use on behavior need to be controlled when investigating iOUD. Furthermore, if EBDM is influenced by substance use-related changes, then there may be synergistic effects of dual opioid and tobacco use in EBDM behavior.

We hypothesize that TDRL will be a better explanation of behavior on the EEfRT and will better predict unobserved EEfRT choice behavior data. Secondarily, we hypothesize that substance use status will differ in TDRL posterior parameters (learning rate, discount factor, and choice temperature) and it will differ in the multivariate combination of TDRL posterior parameters.

## METHODS

### Participants

#### Study 1 (Addicott et al., 2020)

Participants were recruited from Durham, NC (n = 28) and Little Rock, AR (n = 43) between August 2016 and October 2018 and met criteria for current TUD (n = 26, Current TUD), former TUD (n = 22, Former TUD), and no tobacco use (n = 23, Never TUD). Participants were 18–55 years old. Current TUD smoked ≥ 10 cigarettes/day for ≥ 2 years, expired breath carbon monoxide (CO) concentration of ≥ 10 ppm, morning urinary cotinine concentration of > 100 ng/ml, and no regular use of electronic cigarettes/other nicotine/tobacco products. Former TUD smoked ≥ 10 cigarettes/day for ≥ 2 years, reported no tobacco use ≥ 12 months, breath CO ≤ 5 ppm, and urinary cotinine < 100 ng/ml. Never TUD smoked < 50 cigarettes in their lifetime, reported no use of tobacco or nicotine ≥ 6 months, breath CO ≤ 5 ppm, and urinary cotinine < 100 ng/ml. Participants were screened using structured interviews for medical, psychiatric, and drug use history. Exclusion criteria included serious health problems (e.g. liver, lung or heart disease), psychiatric problems (depression, anxiety, obsessive-compulsive disorder, schizophrenia) with current treatment, a history of neurologic disorders, serious head trauma, or drug/alcohol dependence in the past 6 months (other than tobacco). Participants were also excluded if they tested positive for drugs, alcohol, or pregnancy. Participants provided written informed consent, and this protocol was approved by Duke University’s and University of Arkansas for Medical Sciences’ Institutional Review Boards. Computational analysis was conducted at Wake Forest University School of Medicine.

#### Study 2

Participants enrolled in R01 DA039088 (MPI: Oliveto & Mancino; See (Ray et al., 2025)) between April 2018 and February 2021 at the University of Arkansas for Medical Sciences in Little Rock, AR completed the EEfRT. Participants were 18–65 years old, sought treatment for opioid use, fulfilled DSM-5 criteria for moderate to severe OUD, and provided a urine sample negative for benzodiazepines and barbiturates. Of the 44 participants who completed the EEfRT, n = 29 smoked > 5 cigarettes/day and were included in this analysis (TUD+OUD). Exclusion criteria included severe medical, psychiatric, or neurologic conditions, taking drugs that would interact with the study drugs, and physical dependence on drugs other than opioids, tobacco, or cannabis. See Ray et. al (2025) for complete details. Participants who used alternative forms of nicotine/tobacco (n = 7), those who did not use nicotine/tobacco (n = 5), and those who did not use > 5 cigarettes/day (n = 2) were excluded from the analysis due to their small sample sizes. Participants provided written informed consent, and this protocol was approved by University of Arkansas for Medical Sciences’ Institutional Review Boards. Computational analysis was conducted at Wake Forest University School of Medicine.

### Procedure

This analysis combines data from two studies that used identical versions of the EEfRT. Study 1 included Current TUD, Former TUD, and Never TUD while Study 2 included TUD+OUD. While the study procedures were similar, they are described separately below.

#### Study 1

Individuals with TUD (iTUD) were asked to smoke as usual prior completing a 3-h study visit where the EEfRT was administered at the end of the visit. As part of the EEfRT training, study staff read through the instructions with the participants, and then participants completed four practice trials consisting of at least one low-effort and one high-effort task. After addressing any questions, participants completed the EEfRT. Immediately prior to the EEfRT, participants completed the Shiffman-Jarvik Withdrawal Scale (Shiffman & Jarvik, 1976) to assess six common symptoms of nicotine withdrawal: craving, negative affect, arousal, somatic symptoms, appetite, and habit withdrawal. Never TUD participants completed non-craving/habit withdrawal questions to assess general/nonspecific discomfort.

#### Study 2

This study examined adjunct gabapentin during outpatient buprenorphine detoxification to improve OUD treatment outcomes. All data analyzed were collected prior to buprenorphine dosing. At the first study visit, after completing eligibility requirements, participants provided a urine sample and completed assessments including the Subjective Opiate Withdrawal Scale (SOWS), consisting of 16 items describing possible opioid withdrawal symptoms they might be experiencing at the time of survey completion rated on a scale from 0 (not at all) to 4 (extremely) (Handelsman et al., 1987). Then participants completed the EEfRT with instructions read by study staff and completing four practice trials consisting of at least one low-effort and one high-effort task.

### Effort Expenditure for Rewards Task (Treadway et al., 2009)

**Fig. 1:**
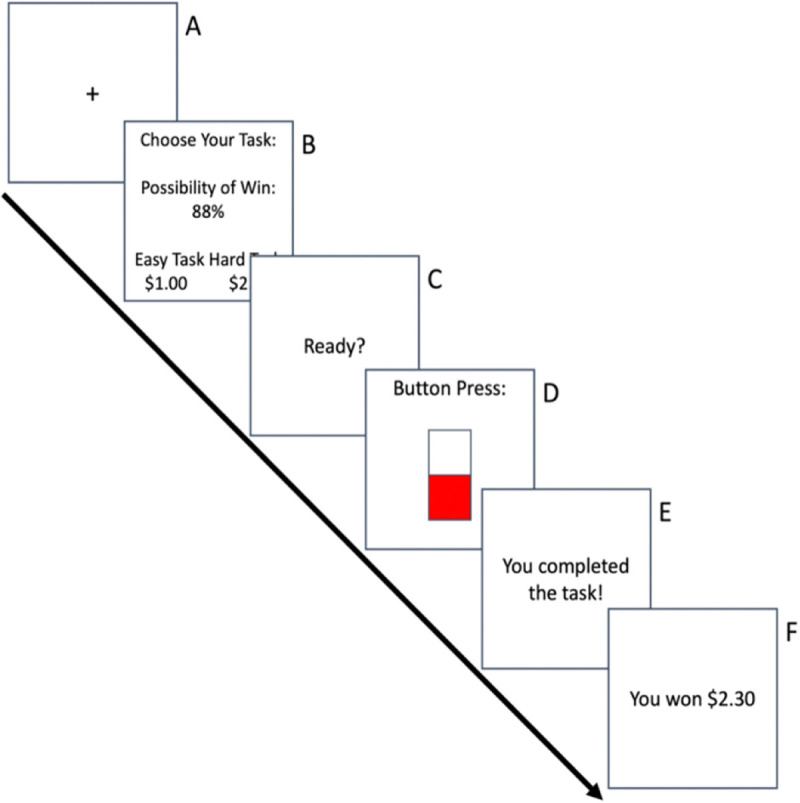
Effort Expenditure for Rewards Task Depiction of a single EEfRT trial. A) 1 sec fixation cue. B) Five sec choice period in which participants are presented with the reward magnitude and probability. C) 1 sec “Ready” screen. D) Action screen where participants make rapid button presses. Easy Task: 30 button presses using the dominant index finger in 7 sec. Hard Task: 100 button presses using the non-dominant pinky finger in 21 sec. E) 2 sec Feedback on completion/incompletion. F) 2 sec Monetary reward feedback.

The effort expenditure for rewards task (EEfRT) is a multi-trial, two-choice, cost-benefit task designed to measure one’s willingness to exert effort (Treadway et al., 2009). Figure 1 depicts a single EEfRT trial. Participants are given the opportunity to choose between two different task difficulty levels, hard and easy, to obtain monetary rewards. Both task options consist of repeated manual button presses within a timeframe. Complete button presses are represented onscreen by the height of a vertical bar. The low-effort, easy task option requires 30 button presses with the dominant index finger within 7 s. The high-effort, hard task option requires 100 button presses with the nondominant little finger within 21 s. Participants are monitored during the task to ensure that they use the correct finger for each task.

In low-effort trials, participants can receive $1.00 if they complete the task on time. In high-effort trials, participants can receive a variable reward between $1.24 and $4.30 (i.e. reward magnitude). Across trials (both low- and high-effort tasks), the likelihood of receiving a reward upon successful completion of the trial is either 12%, 50%, or 88% (i.e. reward probability). At the start of each trial, participants are shown the reward magnitude for both task options and the probability level. Participants have 5 s to make a choice; if a task is not selected, they are randomly assigned to a task. After selecting the task, they complete the button press task and immediately receive feedback informing them if the task is completed successfully and whether they received the reward for that trial. Participants are informed that a single trial that resulted in a reward will be selected at random at the end of the EEfRT and the participant would be given this amount (between $1 and $5) as bonus pay.

Low-effort trials last approximately 15 s and high-effort trials last approximately 30 s. Participants are told they have 20 minutes to play as many trials as possible. They are informed of the trade-off between choosing too many high-effort trials early in the game then missing out on playing large reward magnitude, large probability trials later in the game to discourage the exclusive selection of either the low-effort or high-effort task. Since participants could complete a variable number of trials, only data from the first 50 trials was used for consistency, consistent with previous EEfRT studies (Treadway et al., 2012).

The EEfRT was programmed in MATLAB (Mathworks Inc., Natick, MA) using the Psychtoolbox version 3.0.

### Data Analysis

Prior work examined EEfRT choice behavior in Current, Former, and Never TUD using generalized estimating equation (Addicott et al., 2020). The current secondary analysis extends this work by computationally modeling the dynamics of EEfRT choice behavior in these groups and adding the TUD+OUD cohort to offer insights into group behavior in the use of given information and learning effects in driving choice behavior between the two options.

Participant demographics were analyzed using one-way analysis of variance (ANOVA) with Bonferroni post hoc comparisons for means and chi-square tests for categorical counts. EEfRT performance was analyzed using one-way ANOVA.

To formally test how effort, reward information (magnitude and probability), and feedback drive effort-based choices, we compared two families of computational models, subjective value models and reinforcement learning models.

### Subjective Value Computational Models (Cooper et al., 2019)

#### Full Subjective Value Model

The ‘full subjective value model’ (Full SV model, Eq. 1) will hypothetically fit best for participants who consistently incorporate trial-wise reward and probability information when allocating effort (Cooper et al., 2019). The subjective value of a given trial is calculated by taking the objective reward, R ($1.00 - $4.30), scaling it by the reward probability, P (.12, .50, .88), and reducing it by the amount of effort required to obtain it (0.3 for the low-effort trials or 1.0 for the high-effort trials).

eq. 1 $$SV=R\;*\;P^{h}-kE$$

Free parameters, k and h, capture the individual differences in which reward is discounted by effort and probability of receiving the reward by weighing the components. The 0<k<10 parameter influences subjective value based on the amount of effort required, thus effort that is perceived as costly is reflected by a higher value of k. The 0<h<10 parameter influences subjective value by weighting the probability of receiving the reward.

#### Reward Only Subjective Value Model

Some participants may allocate effort based on reward magnitude alone (Cooper et al., 2019). The reward only subjective value model (Reward Only SV model, Eq. 2) will hypothetically fit best for participants who consistently incorporate trial-wise reward magnitude information but not reward probability information when allocating effort. The subjective value of a given trial is calculated by taking the objective reward, R ($1.00 - $4.30), and reducing it by the amount of effort required to obtain it (0.3 for the low-effort trials or 1.0 for the high-effort trials).

eq. 2 SV=R-kE

Free parameter, k, captures the individual differences in which reward is discounted by effort. The 0<k<10 parameter influences subjective value based on the amount of effort required, thus effort that is perceived as costly is reflected by a higher value of k.

#### Softmax Choice Policy Function

Subjective values (SVs) estimated in the Full SV and Reward Only models are used to estimate choice behavior according to a ‘policy’ function. The policy function defines the strategy for selecting an option based on the current state. These SVs are transformed into probabilities (Eq. 3) of selecting the low-effort or high-effort option using the Softmax choice policy (Sutton & Barto, 1998). This policy function calculates choice probabilities, where the probability, P, of choosing a given option, choice, on each trial, t, was estimated based on its subjective value relative to that of the alternate option. Free parameter 0<β<100 is an inverse choice temperature parameter that captures individual differences in choice stochasticity where it determines the Softmax function slope. Lower inverse temperature values lead to a more randomized choice selection policy and higher inverse temperature values lead to a more exploitative and greedy choice selection policy.

eq. 3 $$P\left({\mathrm{choice}}_{t}=\mathrm{opt}1|SV_{\mathrm{opt}1},SV_{\mathrm{opt}2}\right)=\frac{e^{SV_{\mathrm{opt}1}*\beta }}{e^{SV_{\mathrm{opt}1}*\beta }+e^{SV_{\mathrm{opt}2}*\beta }}$$

### Reinforcement Learning Computational Model (Sutton, 1988; Sutton & Barto, 1998; Watkins & Dayan, 1992)

#### Temporal Difference Reinforcement Learning Model

In model-free temporal difference reinforcement learning (TDRL), agents learn from temporal difference reward prediction errors (TD-RPE, $\delta_{i}$, Eq. 4).

eq. 4 $$\delta_{i}=\left[{\mathrm{outcome}}_{i}+\gamma \;\underset{a}{max}\;Q\left(s_{i+1},\tilde{a}\right)\right]-Q\left(s_{i},a_{i}\right)$$

Here, TD-RPEs are the error between the predicted and actual experiences where outcome $e_{i}$ is the actual outcome (positive or negative) experienced in state $s_{i}$ after taking action $a_{i},\;0<\gamma <1$ is a temporal discount parameter that discounts future expected value, and $\underset{a}{max\;}\;Q\left(s_{i+1},\tilde{a}\right)$ is the maximum expected value over all actions $\tilde{a}$ afforded in the next state $s_{i+1}$.

Following an action-contingent reward or punishment (i.e., $outcome_{i}$), the TD-RPE provides an update value for the current state-action pair, $Q\left(s_{i},a_{i}\right)$, according to a ‘Q-learning’ rule (Eq. 5).

eq. 5 $$Q\left(s_{i},a_{i}\right)\leftarrow Q\left(s_{i},a_{i}\right)+\alpha \delta_{i}$$

Where i indicates discrete time points within a trial and 0<α<1 is the learning rate that parameterizes how much the agent weighs a TD-RPE, $\delta_{i}$, during learning. In the context of the EEfRT, we defined the discrete time points within a trial as i={1,2,3} [1, options presented; 2, action taken; 3, outcome and reward presented]. Also, within the context of the EEfRT, $Q\left(s_{i},a_{i}\right)$ represents the expected value associated with selecting a given option on the ‘Option Presentation’ screen (Fig. 1B) and represents that action’s expected future reward.

#### Softmax Choice Policy Function

As above, we modeled participant choice behavior according to the Softmax choice policy (Sutton & Barto, 1998). This policy function calculates choice probabilities (Eq. 3), whereby the probability, P, of choosing a given option, choice, on each trial, t, was estimated based on its learned Q-value, $Q\left(s_{i},a_{i}\right)$, relative to that of the alternate option.

### Model Fitting

We fit each model using hierarchical Bayesian analysis. Individual level parameter values are drawn from group level distributions over each model parameter. This approach derives individual participant parameter values as deviations from a group mean. For a more detailed description of the hierarchical Bayesian procedure, see (Sands, Jiang, Jones, et al., 2023; Sands, Jiang, Liebenow, et al., 2023). On trials where participants did not make a choice within 5 seconds, they were randomly assigned to a task. In the computational analysis, randomly assigned trials were included for learning updates but excluded from predictive evaluation to reflect all experienced outcomes but avoid biasing predictive accuracy.

All models were fit in Stan using the Hamiltonian Monte Carlo (HMC) sampling algorithm via the R package rstan (version 2.32.6) to estimate the joint posterior distributions for the Subjective Value Models and RL Model across all cohorts simultaneously, with individual parameters hierarchically nested within cohort-level distributions. For all models, we ran four Markov chains, with 10,000 total samples per chain (2,000 warm-up) for a total of 32,000 posterior samples per model parameter. To confirm proper model fitting, we inspected the convergence of the Markov chains by verifying the Gelman-Rubin $\hat{R}$ (i.e., the ratio of within- and between-chain variance), which was approximately or less than 1.1 for almost all parameters across all models.

### Model Comparison

We compared the fit of each model to participant choice behavior on the EEfRT according to model evidence estimates (i.e. Bayesian marginal likelihood), which represents the probability or plausibility of observing the actual EEfRT data under each model (MacKay, 2003). The marginal likelihood for each model is an optimal measure for performing model comparison as it represents the balance between the fit of each model to the data and the complexity of each model. In Bayesian model comparison, the model with the greatest model evidence (i.e. least negative) is deemed the best explanation for the data. We estimated the marginal likelihood for all models using bridge sampling as implemented in the R package bridgesampling [v. 1.1–2; (Gronau et al., 2020)]. Bridge sampling is a statistical technique used to estimate marginal likelihoods of models even with hierarchical structure. We performed 10 repetitions of the sampler and report the median and interquartile range of the estimates of model evidence.

As a secondary comparison, we estimated each model’s Bayesian leave-one-out (LOO) cross-validation predictive accuracy, defined as a model’s expected log predictive density [ELPD-LOO; (Vehtari et al., 2017)]. The ELPD is a statistical technique used to assess how well a statistical model predicts new data by providing an estimate of the predictive accuracy of the model. It focuses on how well the model generalizes to unseen data while penalizing overfitting to the data and it compares models by their predictive performance, helping select the model that balances complexity and fit. The model with the greatest ELPD (i.e. least negative) is deemed the best explanation for the data. We estimated the ELPD for all models for each cohort separately using the R package loo [v. 2.8; (Vehtari et al., 2024)].

### Unsupervised Dimensionality Reduction and Supervised Group Classification

We performed a principal components analysis (PCA) to reduce dimensionality of resulting parameter sets and assess whether distinct covariation amongst the three-vector of TDRL posterior parameter estimates could distinguish between groups. The maximum a posteriori estimate (MAP; the parameter value that maximizes the posterior distribution i.e. the mode of the posterior) of each individual-level TDRL parameter was determined. The resulting three parameters (i.e., learning rate, discount factor, and choice temperature) were concatenated to form a single three-vector per participant and subjected to PCA. Variables were centered and scaled prior to decomposition. Multivariate Analysis of Variance (MANOVA), using Pillai’s trace, was conducted to determine whether groups differ significantly across the multivariate space defined by the combination of all principal components (PC). Post-hoc pairwise comparisons were conducted for each PC using ANOVA with Tukey’s Honest Significance Difference test to identify specific group differences in each PC.

We performed a linear discriminant analysis (LDA) to determine if the latent structure captured by the PCA can predict substance use status. We performed a supervised, leave-one-out-cross-validated LDA using each individual’s three-vector of PCs as the independent set of variables and their substance use status as the dependent categorical variable. Classification performance was evaluated using a confusion matrix, from which overall accuracy, per-group sensitivity (true positive rate), and specificity (true negative rate) were computed. Agreement between predicted and actual group membership exceeded chance levels, as indicated by a Cohen’s kappa of 0.68, reflecting substantial reliability. Receiver operating characteristic (ROC) curves were generated for each group using a one-vs-all approach. Posterior probabilities were compared against binary group labels, and area under the curve (AUC) values were computed using the R package pROC [v. 1.18; (Robin et al., 2011)]. 95% confidence intervals for AUC were estimated via DeLong’s method.

All analyses were conducted in R version 4.2.3.

## RESULTS

Participant demographics and smoking histories are shown in Table 1. Two participants were excluded for not completing at least 50 trials. One participant was excluded for selecting only the easy task option. Groups did not differ in sample size (**χ**^2^(3) = 1.2, p = 0.753), sex distribution (**χ** ^2^(3) = 5.43, p = .143), or cigarettes/day (F(2,74) = 0.40, p = 0.675), although they did significantly differ in age (F(3,96) = 13.88, p < 0.001) and racial distribution (**χ** ^2^(12) = 42.80, p < 0.001). When comparing Shiffman-Jarvik Scores, groups did not differ in negative affect, arousal, somatic symptoms, and appetite but groups did significantly differ in craving (F(1,45) = 53.16, p < 0.001) and habit withdrawal (F(1,45) = 4.258, p = 0.0449) as expected. See Table 1 for results of group comparisons.

### TDRL Model Best Explains Choice Behavior on the EEfRT

We fit the Subjective Value models and TDRL model to participant choice behavior using hierarchical Bayesian inference and compared estimates of the model evidence (i.e. marginal likelihood) and the posterior predictive accuracy (density) for all models. TDRL demonstrated greater model evidence and greater posterior predictive accuracy relative to the Subjective Value Models (Table 2), indicating that TDRL is a better description of behavior on the EEfRT and better predicts EEfRT choice behavior data.

**Fig. 2:**
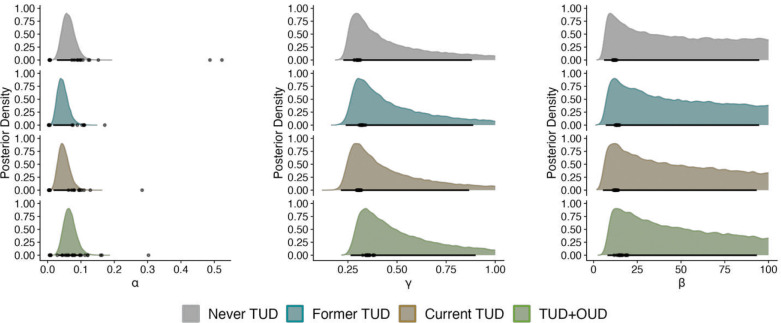
Posterior parameter distributions for TDRL Each subplot displays the distributions for a distinct parameter: α – learning rate; γ – discount factor; β – inverse temperature parameter. Posterior distributions are visualized using slab densities normalized within each panel to facilitate comparison across groups. Group-level distributions are represented in the density plots. Individual-level parameter maximum a posteriori (MAP) estimated by the model are marked by points below the posterior distributions. The 95% highest density interval is indicated by the black line below the distribution.

Given that participant choice behavior is better explained by TDRL, we next investigated group differences in the posterior parameter distributions. The group-level TDRL model parameters are reported in Supplemental Table 1 and Figure 2. Supplemental Figure 1 reports posterior distribution of differences between each and every group. Evidence of group distributional differences is present when the 95% highest density interval (HDI) excluded 0 or was within 0.05 of excluding 0 (Kruschke, 2010). At the group-level there were no credible differences between groups for individual α, γ, and β parameters (Supplemental Figure 1).

### Group Differences in a Dynamic Behavioral Change by Substance Use Status

**Fig. 3:**
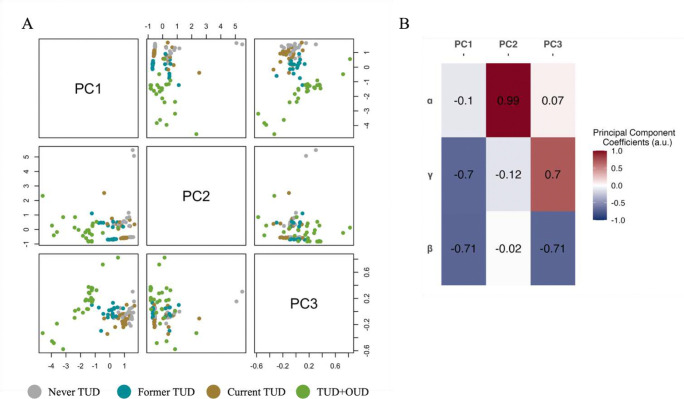
Principal components analysis of TDRL parameters Principal components analysis illustrating group differences in TDRL parameters. (A) PCA pairs plots illustrating biplots for each and every pairing of principal components. Dots indicate individual principal component values. Colors indicate group classification of each individual. (B) Principal component loadings of each TDRL parameter to each principal component. Warm colors indicate a more positive loading and cool colors indicate a more negative loading.

We performed a PCA to reduce dimensionality and assess whether distinct covariation amongst the three-vector of TDRL parameters could distinguish between groups. Each PC explained the following amount of the variance: PC1 = 65.4%, PC2 = 33.2%, and PC3 = 1.5%. Figure 3B details the loading strength (i.e. a coefficient that represents the strength and direction of the relationship) of each TDRL parameter for each PC. PCA revealed meaningful group differences as visualized in Figure 3 and confirmed by MANOVA (F(3,96) = 16.08, p-value < 0.001). Post-hoc pairwise comparisons identified specific group differences in PC1 and PC3 (Supplemental Table 2). For PC1 all group pairings are significantly different excluding Never TUD compared to Current TUD. For PC2, all group pairings are not significantly different. For PC3 TUD+OUD is significantly different from all other groups.

**Fig. 4:**
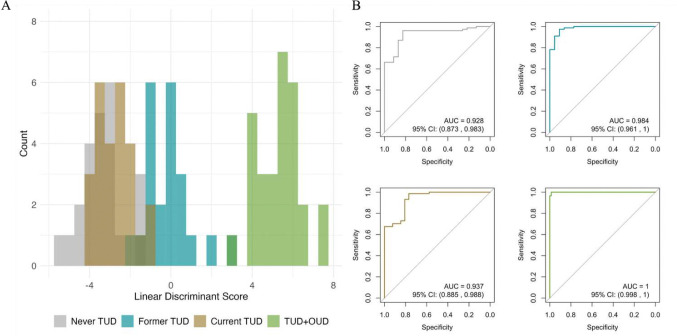
Linear discriminant analysis of substance use Linear discriminant analysis illustrating group classification results based on principal component inputs. (A) LDA histogram of the first linear discriminant dimension colored by group, illustrating group separation across discriminant dimensions. Colors indicate group classification. (B) Receiver operating characteristic (ROC) curves showing comparison of true and false positive rates for group versus not group classification (i.e. Never TUD versus not Never TUD), with associated area under the curve (AUC) and 95% confidence intervals (CI).

We performed a LDA to determine if the latent structure captured by the PCA can classify substance use status. We performed a supervised, leave-one-out-cross-validated LDA using each individual’s three-vector of PCs as the independent set of variables and their known substance use status as the dependent categorical variable. The linear discriminant coefficients (i.e. weights assigned to each PC in forming the linear discriminant axes) is as follows – LD1: PC1 = −2.40, PC2 = −0.51, PC3 = 7.36; LD2: PC1 = −0.11, PC2 = −0.82, PC3 = −2.84; LD3: PC1 = 0.29, PC2 = −0.58, PC3 = 3.31. Table 3 shows the resulting confusion matrix from the LDA. Figure 4A illustrates group separation across the first discriminant dimension. The overall classification accuracy is 82%. Sensitivity (true positive rate) for each group is: Never TUD = 56.5%, Former TUD = 90.9%, Current TUD = 80.8%, and TUD+OUD = 96.6%. Specificity (true negative rate) for each group is: Never TUD = 96.1%, Former TUD = 96.2%, Current TUD = 85.1%, and TUD+OUD = 98.6%. Agreement between predicted and actual group membership exceeded chance levels, as indicated by a Cohen’s kappa of 0.76, reflecting substantial reliability. The resulting ROC curves (Fig. 4B) show excellent diagnostic performance.

## DISCUSSION

EBDM is a critical cost-benefit analysis that has been implicated in psychiatric disorders. Thus, we examined how current and former iTUD and current iOUD make effort-based decisions. As hypothesized, all groups (Never TUD, Former TUD, Current TUD, and TUD+OUD) use a RL framework to make effort-based decisions. Although groups did not differ significantly in TDRL parameters (learning rate, discount factor, and choice temperature), groups differ in their multivariate combination of TDRL posterior parameters, which accurately identifies substance use status.

Groups differ by substance use status in a dynamic behavioral change (i.e. learning) as measured by the multivariate combination of learning rate, discount factor, and choice temperature. PCA revealed potential decision-making dimensions that reliably classified groups. PC1 is the joint dimension of future reward sensitivity and choice stochasticity with lower scores indicating reduced sensitivity to future reward value and more random/less consistent choices. PC1 suggests a.) no difference between Never and Current TUD, b.) steeper discounting of future rewards and more stochastic/random choices characterize Former TUD when compared to Never TUD and Current TUD, and c.) steeper discounting of future rewards and more stochastic/less consistent choices characterize TUD+OUD. PC2, the learning rate dimension, showed no group differences, suggesting similar rates of updating value estimates from feedback. PC3 captures the tradeoff between future reward sensitivity and choice stochasticity with higher scores indicating more sensitivity to future reward value and more random/less consistent choices. PC3 suggests TUD+OUD may be characterized by less discounting of future rewards and more stochastic/random choices. These findings together suggest that TUD+OUD is characterized by overall alteration in decision making with altered choice stochasticity as a distinguishing feature, while steeper discounting of future rewards and increased stochastic/random choices distinguish Former TUD from Never and Current TUD. Further studies are needed to clarify these speculations, particularly with a control group with OUD only and a study design meant to study these intricacies.

EBDM has been studied in both animals and humans using paradigms that offer low-effort/low-reward options and high-effort/high-reward options (Salamone et al., 2007). The EEfRT, adapted from animal paradigms, assesses willingness to exert effort for a monetary reward (Treadway et al., 2009). Extensive research shows that DA transmission modulates effort expenditure (Assadi et al., 2009; Caul & Brindle, 2001; de Jong et al., 2015; Floresco et al., 2008; Kurniawan, 2011; Salamone et al., 2016, 2022; Wardle et al., 2011). Individuals with psychiatric disorders characterized by DA dysregulation perform differently on EBDM tasks (Barch et al., 2014; Cooper et al., 2019; Culbreth et al., 2018; Hartmann et al., 2015; Treadway et al., 2012; Treadway & Zald, 2011). Substance use disorders have also been associated with dysregulated DA function, decreased endogenous DA function in chronic tobacco use (Ashok et al., 2019; Dagher et al., 2001; Fehr et al., 2008) and reduced DA transporter availability in chronic opioid use (Shi et al., 2008), which may lead to differences in EBDM performance. Prior studies have established that TDRL tracks decision-making behavior and underlying dopaminergic mechanisms (Liebenow et al., 2022; Montague et al., 1996; Sands, Jiang, Jones, et al., 2023; Sands, Jiang, Liebenow, et al., 2023; Schultz et al., 1997; Sutton & Barto, 1998) but to our knowledge, no study has shown that the Subjective Value Models track underlying dopaminergic mechanisms. Consistent with this DA modulation framework, we show that TDRL better explains EBDM in healthy controls, iTUD, and iOUD. This preliminary work motivates future work to directly investigate the potentially similar dopaminergic underpinnings of TDRL, EBDM (specifically in the EEfRT), and TUD/OUD.

TDRL, in comparison to the Subjective Value Models, offers a framework to characterize behavioral differences that require multiple trials to observe. TDRL is defined by the ability to learn from interactions with an environment through trial and error to maximize a cumulative reward outcome. It achieves this through its ability to learn predictions of future rewards over time by updating value estimates based on differences between successive predictions. We show that this accumulation of predictive knowledge is critical for modeling EBDM, even in the absence of an explicit effort expenditure parameter.

Few studies have examined tobacco or opioid effects on EBDM, with mixed findings. On a modified EEfRT, iTUD made more high-effort selections during 1 week of abstinence than tobacco-satiated baseline, though this could be attributed to a learning effect (Hughes et al., 2017). Smoking status has been shown to affect EBDM, with current tobacco use associated with diminished reward sensitivity and former tobacco use with greater reward sensitivity (Addicott et al., 2020). Other studies have reported no effects of smoking status or tobacco withdrawal on physical effort discounting for money (S. Mitchell, 2004; S. H. Mitchell, 1999). Together, these findings suggests a relationship between EBDM and tobacco use.

Work by Jarvis et. al (2022) applied Rescorla-Wagner RL models to an EBDM paradigm, which combined probabilistic reward with physical effort, linking effort to learning by examining how effort modulated learning rates (Jarvis et al., 2022). Future work could extend TDRL to incorporate effort effects. Also, TDRL has been extended through Valence Partitioned Reinforcement Learning (VPRL), which partitions appetitive (Positive Valence) and aversive (Negative Valence) information into independent, parallel reward and punishment specific value systems with distinct prediction errors (Kishida & Sands, n.d.; Sands, Jiang, Jones, et al., 2023; Sands, Jiang, Liebenow, et al., 2023). Future work should investigate if VPRL distinguishes reward/punishment (cost/benefit) learning better in EBDM.

This study has several limitations. Groups differed in age, years of education, and race which were not controlled in the current modeling approach. However, we examined associations between TDRL posterior parameters and demographic metrics (see Supplement). Secondly, lower high-effort selections than in other community-based samples, potentially reducing generalizability (Barch et al., 2014; Huang et al., 2016). An OUD participant group was not included, however TUD co-morbidity is more prevalent than exclusive opioid use, as it is estimated 70–85% of individuals with OUD use tobacco (John et al., 2019; McHugh et al., 2020; Parker et al., 2020). Also, this analysis combined two studies which brings limitations of geographical location and collection date. Lastly, while results are consistent with dopaminergic accounts of effort, this interpretation remains speculative and direct investigation is needed.

In summary, effort-based decisions on the EEfRT are made using a RL framework and a dynamic behavioral change (i.e. learning) as measured by the multivariate combination of learning rate, discount factor, and choice temperature differed by substance use status. Future studies should further investigate effort within RL frameworks and clarify dopaminergic function, EBDM, and substance use.

## Supplementary Material

Supplementary Files

This is a list of supplementary files associated with this preprint. Click to download.

• GBNDITOPaperSupp.docx

## Figures and Tables

**Table 1.** Participant Demographics and Smoking Histories. Values reported are either categorical counts (n, Sex, and Race) or mean ± standard deviation. Between-group comparisons were performed using one-way analysis of variance (ANOVA) with Bonferroni post hoc comparisons for means and chi-square tests for categorical counts. Bold p-values are significant.

	Never TUD (n = 23)	Former TUD (n = 22)	Current TUD (n = 26)	TUD+OUD (n = 29)	Significance (p value)

Sex (F/M)	12 / 11	11 / 11	18 / 8	11 / 18	χ^2^ (3) = 5.43, p = .143
Age	28.7 ± 8.9	38.5 ± 8.3	41.8 ± 10.2	30.5 ± 6.0	**F(3,96) = 13.88, p < 0.001 Never TUD < Former TUD & Current TUD, TUD+OUD < Former TUD & Current TUD**
Years of education	15.7 ± 2.2	14.9. ± 2.7	13.5 ± 2.0	13.5 ± 1.5	**F(3,96) = 6.40, p < 0.001 Never TUD > Current TUD & TUD+OUD**
Race					**χ^2^ (12) = 42.80, p < 0.001**
Asian	5	1	0	0	**Asian Never TUD > all other groups**
American Indian	0	0	0	1	**Black Current TUD > TUD+OUD**
Black	8	4	16	1	**White TUD+OUD > Never TUD & Current TUD**
White	10	17	10	26	
Other	0	0	0	1	
Cigarettes/Day		17.2 ± 11.0	16.6 ± 10.3	14.9 ± 8.7	F(2,74) = 0.40, p = 0.675
Shiffman-Jarvik Craving		2.0 ± 1.3	5.0 ± 1.4		**F(1,45) = 53.16, p < 0.001 Former TUD < Current TUD**
Shiffman-Jarvik Negative Affect	3.5 ± 0.9	3.8 ± 1.4	4.0 ± 1.1		F(2,68) = 1.352, p = 0.265
Shiffman-Jarvik Arousal	1.8 ± 1.1	2.0 ± 1.3	1.9 ± 1.1		F(2,68) = 0.004, p = 0.996
Shiffman-Jarvik Somatic Symptoms	5.4 ± 0.9	5.3 ± 0.9	5.4 ± 0.9		F(2,68) = 0.154, p = 0.858
Shiffman-Jarvik Appetite	1.4 ± 0.4	1.4 ± 0.4	1.4 ± 0.3		F(2,68) = 0.312, p = 0.733
Shiffman-Jarvik Habit Withdrawal		2.0 ± 1.5	2.8 ± 1.2		**F(1,45) = 4.258, p = 0.0449 Former TUD < Current TUD**
SOWS Total Score				9.6 ± 9.9	

**Table 2.** Effort-based and RL Model Comparison results. Computed estimates of the Bayesian model evidence (i.e., marginal likelihood via bridge sampling) and model predictive density (i.e., expected log predictive density) for Effort-based and RL models. Reported values are the estimate value with interquartile range (of model evidence) and standard error reported (for predictive density) in parentheses. TDRL demonstrated the maximum (least negative) model evidence and predictive density compared to the Effort-based models.

	All Participants	

Model	Model Evidence	Predictive Density
Full SV	−3222.3 (0.03)	−3442.4 (5.40)
Reward Only	−3319.5 (0.02)	−3442.3 (5.41)
TDRL	**−2952.3 (0.72)**	**−3167.4 (48.88)**

**Table 3.** Linear discriminant analysis confusion matrix. Reported are the actual versus predicted group classification from leave-one-out cross validation linear discriminant analysis. Diagonal cells indicate correct classifications. The overall classification accuracy is 82%.

		Actual				
		Never TUD	Former TUD	Current TUD	TUD+OUD	Totals

**Predicted**	Never TUD	13	0	3	0	16
	Former TUD	0	20	2	1	23
	Current TUD	10	1	21	0	32
	TUD+OUD	0	1	0	28	29
	Totals	23	22	26	29	100
